# Continuous glucose monitoring in pregnant women with type 1 diabetes: an observational cohort study of 186 pregnancies

**DOI:** 10.1007/s00125-019-4850-0

**Published:** 2019-03-23

**Authors:** Karl Kristensen, Linda E. Ögge, Verena Sengpiel, Karin Kjölhede, Annika Dotevall, Anders Elfvin, Filip K. Knop, Nana Wiberg, Anastasia Katsarou, Nael Shaat, Lars Kristensen, Kerstin Berntorp

**Affiliations:** 1 Department of Clinical Sciences Lund, Lund University, Sölvegatan 19, 221 84 Lund, Sweden; 2 The Parker Institute, Copenhagen University Hospital, Copenhagen, Denmark; 3 Department of Obstetrics and Gynecology, Skåne University Hospital, Malmö, Sweden; 4 Department of Obstetrics and Gynecology, Sahlgrenska University Hospital, Gothenburg, Sweden; 5 Sahlgrenska Academy, University of Gothenburg, Gothenburg, Sweden; 6 Department of Medicine, Östra/Sahlgrenska University Hospital, Gothenburg, Sweden; 7 Department of Pediatrics, Sahlgrenska University Hospital, Gothenburg, Sweden; 8 Clinical Metabolic Physiology, Steno Diabetes Center Copenhagen, Gentofte Hospital, Hellerup, Denmark; 9 Department of Clinical Medicine, Faculty of Health and Medical Sciences, University of Copenhagen, Copenhagen, Denmark; 10 Department of Endocrinology, Skåne University Hospital, Malmö, Sweden; 11 Department of Clinical Sciences Malmö, Lund University, Lund, Sweden

**Keywords:** Continuous glucose monitoring, Fetal growth, Neonatal complications, Pregnancy, Type 1 diabetes

## Abstract

**Aims/hypothesis:**

The aim of this study was to analyse patterns of continuous glucose monitoring (CGM) data for associations with large for gestational age (LGA) infants and an adverse neonatal composite outcome (NCO) in pregnancies in women with type 1 diabetes.

**Methods:**

This was an observational cohort study of 186 pregnant women with type 1 diabetes in Sweden. The interstitial glucose readings from 92 real-time (rt) CGM and 94 intermittently viewed (i) CGM devices were used to calculate mean glucose, SD, CV%, time spent in target range (3.5–7.8 mmol/l), mean amplitude of glucose excursions and also high and low blood glucose indices (HBGI and LBGI, respectively). Electronic records provided information on maternal demographics and neonatal outcomes. Associations between CGM indices and neonatal outcomes were analysed by stepwise logistic regression analysis adjusted for confounders.

**Results:**

The number of infants born LGA was similar in rtCGM and iCGM users (52% vs 53%). In the combined group, elevated mean glucose levels in the second and the third trimester were significantly associated with LGA (OR 1.53, 95% CI 1.12, 2.08, and OR 1.57, 95% CI 1.12, 2.19, respectively). Furthermore, a high percentage of time in target in the second and the third trimester was associated with lower risk of LGA (OR 0.96, 95% CI 0.94, 0.99 and OR 0.97, 95% CI 0.95, 1.00, respectively). The same associations were found for mean glucose and for time in target and the risk of NCO in all trimesters. SD was significantly associated with LGA in the second trimester and with NCO in the third trimester. Glucose patterns did not differ between rtCGM and iCGM users except that rtCGM users had lower LBGI and spent less time below target.

**Conclusions/interpretation:**

Higher mean glucose levels, higher SD and less time in target range were associated with increased risk of LGA and NCO. Despite the use of CGM throughout pregnancy, the day-to-day glucose control was not optimal and the incidence of LGA remained high.

**Electronic supplementary material:**

The online version of this article (10.1007/s00125-019-4850-0) contains peer-reviewed but unedited supplementary material, which is available to authorised users.

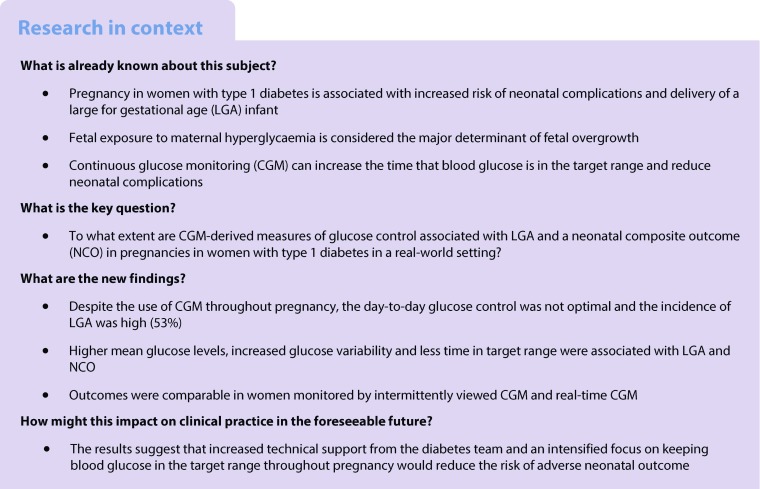



## Introduction

Despite improved glycaemic control, the prevalence of macrosomia and large for gestational age (LGA) remains high in babies born to women with type 1 diabetes, affecting approximately one-half of these newborn infants [1–3]. In addition to an increased risk of obstetric and neonatal adverse outcomes [4], LGA infants have an increased risk of developing obesity, diabetes and cardiovascular disease in later life [5–8].

Fetal exposure to maternal hyperglycaemia is thought to be the major determinant of fetal overgrowth in pregnancies in women with type 1 diabetes [9]. Thus, the overarching goal of prenatal care in these women is to achieve near normal glycaemic control, usually estimated by self-monitoring of plasma glucose and HbA_1c_. However, HbA_1c_ may not adequately reflect fetal glycaemic exposure as it represents an average measure of glycaemic control in the preceding 2–3 months and does not capture acute glucose fluctuations or intra- and inter-day glycaemic variability [10–12]. Moreover, tight glycaemic control may be difficult to accomplish, given the complexity of insulin dose adjustment required to account for gestational changes in insulin sensitivity and variability in insulin absorption during pregnancy [13, 14]. Recent data have shown that fewer than 50% of pregnant women with diabetes in the UK reach target HbA_1c_ levels [15].

Continuous glucose monitoring (CGM) technology provides unique insights into daily glycaemic control and permits a better understanding of how glycaemic patterns and glucose variability may influence pregnancy outcomes. The effectiveness of intermittent use of CGM in pregestational diabetes (type 1 diabetes and type 2 diabetes) in improving glycaemic control and reducing the risk of macrosomia has been evaluated in two randomised controlled trials, in the UK and Denmark, with conflicting results [16, 17]. Merged data from the two studies showed that LGA was associated with trimester-specific differences in daily glucose patterns, i.e. with lower mean glucose and less glycaemic variability in the first trimester and with higher mean glucose and more variable glucose levels in the second and third trimesters [18]. Other groups have similarly shown that higher glycaemic variability, especially during late pregnancy, may increase the risk of LGA [19, 20]. A more recent trial, CONCEPTT, found that continuous use of real-time CGM in pregnancies in women with type 1 diabetes resulted in greater reduction in HbA_1c_, more time spent in the target range, less time spent above the target range and reduced glucose variability. Furthermore, neonatal outcomes were improved, including a lower incidence of LGA infants and a decrease in neonatal hypoglycaemia [21]. The extent to which the CGM-derived measures of glucose control are associated with LGA in a clinical setting is, however, unclear.

In our regions in southwestern Sweden, women with type 1 diabetes are offered a CGM device as part of routine pregnancy care. Here, we report CGM summary data from a cohort of Swedish women who received pregnancy care during the years 2014 to 2017, using the recently published international consensus recommendation for optimal analysis of CGM data [22]. The aim of the study was to determine patterns of maternal glucose control during different phases of pregnancy and to examine whether these patterns are associated with LGA and a predefined adverse neonatal composite outcome (NCO).

## Methods

### Study population

We performed a retrospective analysis of CGM data in women with type 1 diabetes who received pregnancy care between 2014 and 2017 at two large tertiary care antenatal clinics in Sweden (Skåne University Hospital and Östra/Sahlgrenska University Hospital). All women above 18 years of age using a CGM device compatible with the internet-based Diasend system (Glooko, Gothenburg, Sweden) were eligible for inclusion in the study. CGM data were available from 192 women. Of these, three women decided to opt out. Another three women were excluded because of: termination of pregnancy due to chromosome aberration (*n* = 1); intrauterine fetal demise (*n* = 1); and multiple gestation (*n* = 1). After exclusion of these pregnancies, CGM data from 186 singleton pregnancies were available for analysis.

### Management of diabetes in pregnancy

All women received routine clinical care, with antenatal visits every 2 to 4 weeks. In Sweden, the use of CGM is reimbursed in type 1 diabetes outside of pregnancy if adequate glucose control is not achieved by conventional methods, and for all women in pregnancy. Women who were not already using a CGM device before pregnancy (*n* = 84) were offered one at the first antenatal clinic visit, either real-time (rt) CGM or intermittently viewed (i) CGM (*n* = 102). The women made their own choice of which CGM device to use. In all, 40 women declined or did not get along with CGM throughout pregnancy. Moreover, 45 women used CGM devices or pumps not compatible with the Diasend system (Medtronic). In addition to CGM, self-monitored plasma glucose measurements were recommended at a minimum frequency of twice daily. Treatment goals for glucose were <6 mmol/l before meals, <8 mmol/l 1 h after meals and 6–8 mmol/l before bedtime. All glucose values were downloaded to the Diasend system on a weekly basis and the results were communicated to a diabetologist or a trained diabetes nurse for adjustment of insulin doses. HbA_1c_ was measured every 4 to 8 weeks during pregnancy and the mean value for each trimester was calculated. HbA_1c_ analysis was performed according to the International Federation of Clinical Chemistry standards, with measurement in mmol/mol and conversion to % levels according to the National Glycohemoglobin Standardization Program for dual reporting.

### CGM system

The CGM device used was either Dexcom G4 (Dexcom, San Diego, CA, USA) or Freestyle Libre (Abbott Diabetes Care, Alameda, CA, USA), which are both compatible with the Diasend system. The Dexcom G4 system, hereon referred to as rtCGM, measures subcutaneous interstitial glucose concentration every 10 s and generates a glucose value every 5 min (with 288 recordings per day). The monitor requires calibration by the user against capillary plasma glucose twice a day. The Freestyle Libre system, hereon referred to as iCGM, uses a similar method to show continuous glucose measurements retrospectively at the time of checking. It uploads the glucose level every 60 s and generates a glucose value every 15 min (with 96 recordings per day). The device requires no calibration by the user. An important difference between the two systems is that rtCGM has an alarm that warns the user if the glucose is trending towards hypoglycaemia or hyperglycaemia. In all, 38% of iCGM users were CGM naive as opposed to 72% of rtCGM users.

### CGM data management

The raw downloaded CGM dataset was stratified for gestational day and week using Microsoft Access software (Microsoft 2015, Redmond, WA, USA). The dataset for each pregnancy was split into 14-day periods and trimesters (gestational weeks <13, 13–28 and >28). We followed the recently published consensus on use of CGM and required that there were a minimum of 14 consecutive days of data with at least 80% coverage for inclusion [22].

### CGM metrics

We calculated a range of summary statistical CGM indices from the raw downloaded glucose data, including mean CGM glucose level and the percentage of time spent within, below and above the pregnancy glucose target range (3.5–7.8 mmol/l). Measures of glycaemic variability included the following: SD of mean glucose; CV%; mean amplitude of glucose excursions (MAGE), which summarises glycaemic variability by identifying and summarising significant glucose peaks and nadirs for which amplitude exceeds one SD [23]; high blood glucose index (HBGI) and low blood glucose index (LBGI), which convert glucose values into risk scores around zero―predicting the risk of high and low glucose values, respectively [24]. For the calculation of MAGE, we used the algorithm described by Baghurst [25] but did not include amplitudes if the missing values exceeded 60 min [26]. The HBGI and LBGI are derived from a logarithmic transformation of the blood glucose scale that balances the amplitude of hypoglycaemic and hyperglycaemic ranges (enlarging the former and shrinking the latter) and makes the transformed data symmetric around zero―fitting a normal distribution [24]. For the calculation of HBGI and LBGI, we used the formulae described by Fabris et al [27].

### Obstetric data and outcomes

Electronic antenatal and perinatal records provided data on maternal age, parity, BMI, country of origin, HbA_1c_ levels, duration of diabetes, insulin regimen (i.e. insulin pump or multiple daily injections), mode of delivery, birthweight, gestational age at birth and sex of infant. In addition, information about pre-eclampsia or pregnancy-induced hypertension was obtained. All pregnancies were dated by ultrasound examination before 22 weeks of gestation. LGA was defined as birthweight >2 SD above the expected birthweight for gestational age and sex, respectively, according to the Swedish reference curve for fetal growth [28]. Macrosomia was defined as birthweight >4500 g. Neonatal complications, such as macrosomia, shoulder dystocia, neonatal hypoglycaemia (defined as plasma glucose <2.6 mmol/l >3 h after birth) or admission to the neonatal intensive care unit (NICU) for more than 24 h, were recorded. The main neonatal outcomes were LGA, and NCO that included at least one of: macrosomia; shoulder dystocia; neonatal hypoglycaemia; or admission to NICU for more than 24 h.

### Ethical considerations

The study was approved by the Ethics Committee of Lund University (2017/322) and was conducted in accordance with the Swedish Act on Ethics Review of Research Involving Humans and the Swedish Act on Personal Data. All women who were included received written information about the study and gave informed consent.

### Statistics

Differences in means were tested with unpaired *t* test and differences in medians were tested with the Mann–Whitney *U* test. Frequencies were compared using the *χ*^2^ test. The (fixed) effect(s) of gestational age were analysed by repeated-measures mixed-model analysis (linear, compound symmetry). Differences in glucose outcome(s) between women monitored by either rtCGM or iCGM were analysed by one-way repeated-measures ANOVA. Associations between the glucose indices and neonatal outcomes were analysed by stepwise (hierarchal) logistical regression analysis with and without adjusting for confounders. The regression model was adjusted for maternal age, smoking, early-pregnancy BMI, and CGM device. The regression models were not adjusted for the intermediate variables HbA_1c_, gestational age or maternal gestational weight gain [29]. Preterm deliveries before 34 weeks were excluded from the model testing for associations with the NCO. Missing data were below 5% for all variables. Two-sided *p* values <0.05 were considered to be statistically significant. IBM SPSS Statistics version 24.0 for Windows (IBM Corporation, Armonk, NY, USA) was used for all analyses. Based on the size of the cohort and the volume of CGM data available, our analyses had 80% power at the 5% level to detect a 0.4 mmol/l difference in mean glucose concentration between participants who delivered infants with or without LGA.

## Results

### Measurements in analysis

Data from the 186 singleton pregnancies (105 from Skåne University Hospital and 81 from Östra/Sahlgrenska University Hospital) with at least one 2-week episode with 80% coverage were available for analysis. Altogether, the dataset comprised 2944 2-week episodes. After the exclusion of 638 2-week episodes with less than 80% coverage, 5.75 million glucose measurements conducted over 2306 separate measurement episodes were available for the analysis. The excluded CGM profiles (32% rtCGM and 12% iCGM) were evenly spread across trimesters. Of the 186 women, 155 (83%) had measurement episodes in the first trimester, 165 (89%) in the second trimester and 167 (90%) in the third trimester. Electronic supplementary material (ESM) Table 1 shows the number of women and the number of measurements made in the total cohort according to the glucose monitoring system used.

### rtCGM vs iCGM

**Fig. 1:**
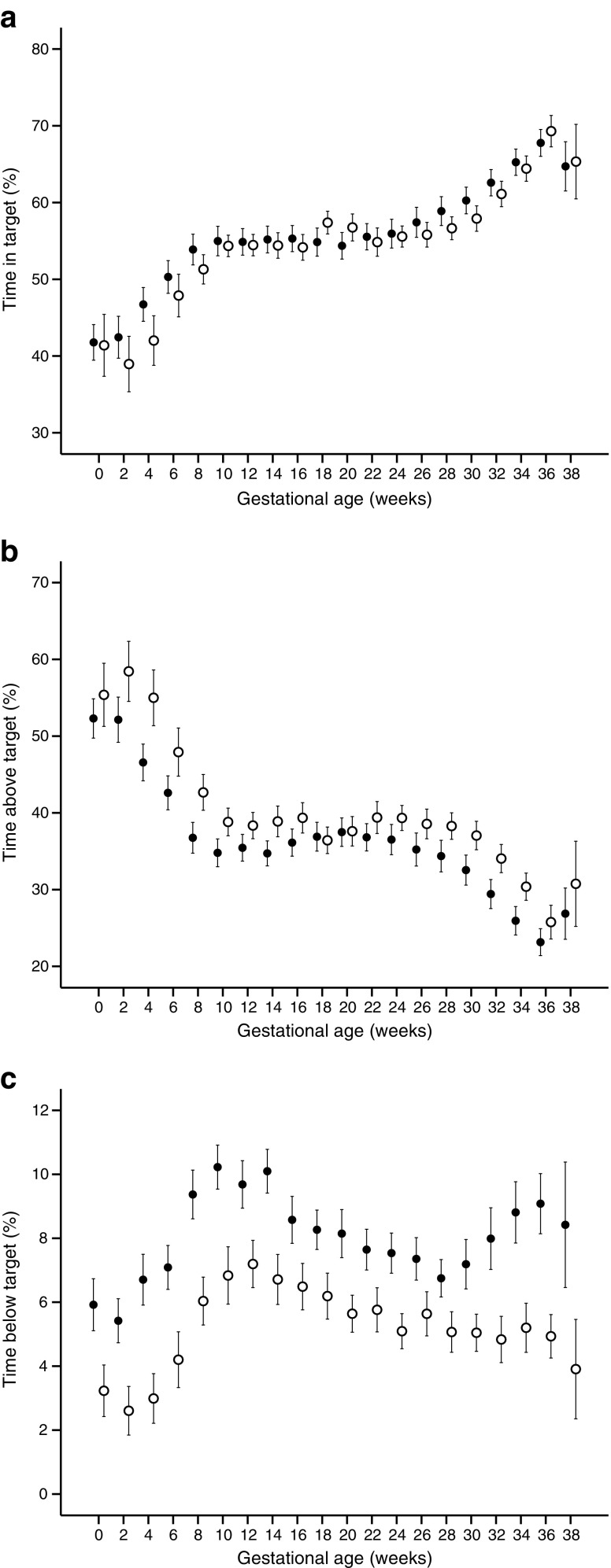
Mean ± SEM of time (%) in (**a**), above (**b**) and below (**c**) the target glucose range (3.5–7.8 mmol/l) in women with type 1 diabetes monitored by rtCGM (white) and iCGM (black) during pregnancy

Mean (SD) values of all the calculated glucose indices for women monitored by either rtCGM or iCGM are shown in ESM Table 2. Figure 1 (a–c) illustrates changes in the proportion of time spent in euglycaemia, hyperglycaemia and hypoglycaemia throughout gestation in the respective CGM group. There were no trimester-specific differences in the proportions of time spent in euglycaemia (*p* = 0.54–0.65) or in hyperglycaemia (*p* = 0.12–0.18). However, women monitored by rtCGM spent less time in hypoglycaemia compared with iCGM users (*p* = 0.006 in the first trimester and *p* = 0.004 in the second and third trimesters). Likewise, the LBGI was significantly lower in all trimesters in women monitored by rtCGM (*p* < 0.001). There were no significant differences for mean glucose levels, SD, CV%, MAGE or HBGI between the two groups (ESM Table 2). There was a clear trend of improved glucose control with increasing gestational age for all the glucose indices in the combined group of women monitored by either rtCGM or iCGM (*p* < 0.001, fixed effect, linear mixed model).

As our intention was to evaluate data pooled from the two CGM systems, we analysed differences in clinical characteristics and outcomes between women using rtCGM and women using iCGM. As shown in Table 1, the maternal characteristics were comparable, with two exceptions. Insulin pumps were used more commonly by women with rtCGM (42% as opposed to 16%). Users of rtCGM also had a longer duration of diabetes. Mean HbA_1c_ levels during pregnancy (52 mmol/mol [6.9%] in the first trimester, and 45 mmol/mol [6.3%] in the second and third trimesters) indicated that overall the women had acceptable glucose control. The proportion of women who achieved a target HbA_1c_ level of <48 mmol/mol (6.5%) in early pregnancy was 37% and this increased to 71% and 68% in the second and third trimesters, respectively, with no significant difference between women using rtCGM and iCGM.

**Table 1:** Maternal characteristics according to glucose monitoring system

Characteristic	Total (n = 186)	rtCGM (n = 92)	iCGM (n = 94)	*p* value
Age, years	31 (19–44)	31 (19–41)	31 (21–44)	0.90
Smokers	21 (11)	8 (9)	13 (14)	0.27
European descent	170 (91)	85 (92)	85 (90)	0.80
Diabetes duration, years	15 (1–34)	17 (2–32)	14 (1–34)	<0.05
Insulin pump	54 (29)	39 (42)	15 (16)	<0.001
Primipara	88 (47)	45 (49)	43 (46)	0.67
Early-pregnancy BMI, kg/m^2^	25.9 ± 4.7	26.4 ± 4.8	25.3 ± 4.5	0.09
Gestational weight gain, kg	14.3 ± 5.5	14.9 ± 6.6	13.8 ± 4.2	0.17
HbA_1c_				
Trimester 1, mmol/mol (%)	52.4 ± 10.5 (6.9 ± 1.0)	52.5 ± 11.1 (7.0 ± 1.0)	52.3 ± 9.8 (6.9 ± 0.9)	0.90
Trimester 2, mmol/mol (%)	45.2 ± 7.9 (6.3 ± 0.7)	45.0 ± 7.6 (6.3 ± 0.7)	45.3 ± 8.3 (6.3 ± 0.8)	0.83
Trimester 3, mmol/mol (%)	45.7 ± 7.6 (6.3 ± 0.7)	45.6 ± 7.7 (6.3 ± 0.7)	45.8 ± 7.5 (6.3 ± 0.7)	0.84

Results are given as *n* (%), mean ± SD or median (range) Missing data were below 5% for all variables

As shown in Table 2, there was no significant difference in maternal and neonatal outcomes between women using rtCGM and women using iCGM. The median gestational age at delivery was 38 weeks with a Caesarean section rate of 47% and an LGA rate of 53%. The proportion of women with LGA offspring did not differ between the two sites of inclusion (*p* = 0.8).

**Table 2:** Maternal and neonatal outcomes according to glucose monitoring system

Outcome	Total cohort (n = 186)	rtCGM (n = 92)	iCGM (n = 94)	*p* value
Pre-eclampsia/PIH	34 (18)	15 (16)	19 (20)	0.47
Caesarean section	87 (47)	46 (50)	41 (44)	0.38
Gestational age (weeks)	38 (27–40)	38 (27–40)	38 (29–40)	0.47
Preterm birth (<37 weeks)	52 (28)	24 (26)	28 (30)	0.57
Female infant	87 (47)	48 (52)	39 (41)	0.14
Birthweight, g	3823 ± 711	3812 ± 678	3834 ± 747	0.84
LGA infant	98 (53)	48 (52)	50 (53)	0.89
Macrosomia (>4500 g)	30 (16)	14 (15)	16 (17)	0.74
5 min Apgar score <7	6 (3)	1 (1)	5 (5)	_
Shoulder dystocia	5 (3)	3 (3)	2 (2)	_
Neonatal hypoglycaemia^a^	45 (24)	19 (21)	26 (28)	0.27
NICU admission >24 h	60 (32)	27 (29)	33 (35)	0.40
NCO^b^	83 (45)	37 (40)	46 (49)	0.23

Results are given as *n* (%), mean ± SD or median (range)

^a^Defined as plasma glucose < 2.6 mmol/l > 3 h after birth

^b^Neonatal composite including ≥ 1 of the following: macrosomia (> 4500 g), shoulder dystocia, neonatal hypoglycaemia or NICU admission > 24 h

Missing data were below 5% for all variables

PIH, pregnancy-induced hypertension

### Glycaemic measures in relation to outcomes

Glycaemic measures recorded and calculated separately for the total cohort and for women who delivered LGA infants and women who delivered non-LGA infants in each trimester are presented in Table 3. Overall, the mean glucose level, SD, CV%, percentage time in hyperglycaemia, MAGE and HBGI decreased in each trimester, whereas the time spent in target increased. This improvement in glucose control was not reflected in HbA_1c_ levels in the latter part of pregnancy. However, the occurrence of LGA was significantly associated with the HbA_1c_ levels in all trimesters. The mean glucose levels and the percentage time spent below, in and above the target range were significantly associated with LGA in the second and third trimesters. Furthermore, LGA was associated with a significantly higher SD of mean glucose in the second trimester.

**Table 3:** Results of the binary logistic regression analysis of variables tested for associations with LGA

Variable	All (n = 186)	LGA (n = 98)	No LGA (n = 88)	Crude data		Adjusted data	
				OR (95% CI)	*p* value	OR (95% CI)	*p* value
Trimester 1 (*n* = 155)							
HbA_1c_, mmol/mol	52.4 ± 10.5	54.1 ± 1.0	50.4 ± 9.5	1.04 (1.00, 1.07)	0.03	1.04 (1.00, 1.08)	0.02*
HbA_1c_, %	6.9 ± 1.0	7.1 ± 1.0	6.8 ± 0.9				
Mean glucose, mmol/l	7.8 ± 1.4	7.9 ± 1.3	7.7 ± 1.5	1.12 (0.88, 1.41)	0.35	1.16 (0.91, 1.49)	0.24
SD, mmol/l	3.2 ± 0.9	3.2 ± 0.8	3.2 ± 0.9	1.07 (0.74, 1.55)	0.71	1.09 (0.73, 1.62)	0.67
CV%	40.5 ± 7.2	40.5 ± 7.2	40.6 ± 7.3	1.00 (0.95, 1.04)	0.90	0.99 (0.95, 1.04)	0.77
Time in target, %^a^	50.0 ± 14.1	48.2 ± 13.6	51.9 ± 14.5	0.98 (0.96, 1.00)	0.10	0.98 (0.95, 1.00)	0.07
Time above target, %	43.0 ± 15.5	44.8 ± 14.6	40.9 ± 16.3	1.02 (0.99, 1.04)	0.11	1.02 (1.00, 1.04)	0.07
Time below target, %	7.0 ± 5.0	7.0 ± 5.1	7.2 ± 5.0	0.99 (0.93, 1.06)	0.81	0.98 (0.92, 1.05)	0.60
MAGE	7.7 ± 2.0	7.8 ± 1.8	7.5 ± 2.1	1.08 (0.92, 1.27)	0.37	1.09 (0.92, 1.30)	0.33
HBGI	5.5 ± 3.7	5.6 ± 3.4	5.3 ± 4.1	1.02 (0.94, 1.11)	0.60	1.04 (0.94, 1.13)	0.46
LBGI	2.6 ± 1.6	2.6 ± 1.6	2.7 ± 1.6	0.97 (0.79, 1.18)	0.74	0.94 (0.76, 1.16)	0.54
Trimester 2 (*n* = 165)							
HbA_1c_, mmol/mol	45.2 ± 7.9	46.4 ± 7.4	43.7 ± 8.3	1.05 (1.00–1.09)	0.03	1.05 (1.01–1.10)	0.02*
HbA_1c_, %	6.3 ± 0.7	6.4 ± 0.7	6.1 ± 0.8)				
Mean glucose, mmol/l	7.4 ± 1.2	7.6 ± 1.0	7.1 ± 1.3	1.48 (1.10, 1.98)	<0.01	1.53 (1.12, 2.08)	<0.001*
SD, mmol/l	2.8 ± 0.7	2.9 ± 0.6	2.7 ± 0.7	1.58 (0.98, 2.56)	0.06	1.65 (1.00, 2.74)	<0.05*
CV%	37.7 ± 6.3	37.8 ± 5.9	37.7 ± 6.7	1.00 (0.95, 1.05)	0.92	1.00 (0.95, 1.06)	0.93
Time in target, %^a^	54.7 ± 13.7	51.8 ± 12.3	57.9 ± 14.4	0.96 (0.94, 0.99)	<0.01	0.96 (0.94, 0.99)	<0.01*
Time above target, %	38.1 ± 14.9	41.9 ± 12.8	34.0 ± 15.9	1.04 (1.02, 1.06)	<0.001	1.04 (1.02, 1.07)	<0.001*
Time below target, %	7.2 ± 5.1	6.4 ± 4.5	8.0 ± 5.7	0.94 (0.88, 0.99)	0.04	0.93 (0.87, 0.99)	0.02*
MAGE	6.8 ± 1.5	7.0 ± 1.4	6.6 ± 1.6	1.21 (0.98, 1.48)	0.07	1.22 (0.98, 1.52)	0.06
HBGI	4.0 ± 2.9	4.4 ± 2.4	3.6 ± 3.3	1.11 (0.98, 1.24)	0.08	1.12 (0.99, 1.26)	0.07
LBGI	2.7 ± 1.6	2.4 ± 1.4	3.0 ± 1.8	0.80 (0.66, 0.97)	0.02	0.77 (0.62, 0.94)	0.01*
Trimester 3 (*n* = 167)							
HbA_1c_, mmol/mol	45.7 ± 7.6	47.2 ± 6.7	44.0 ± 8.2	1.06 (1.02, 1.11)	<0.01	1.06 (1.02, 1.11)	<0.01*
HbA_1c_, %	6.3 ± 0.7	6.5 ± 0.6	6.2 ± 0.8				
Mean glucose, mmol/l	7.1 ± 1.1	7.3 ± 1.1	6.8 ± 1.1	1.57 (1.13, 2.16)	<0.01	1.57 (1.12, 2.19)	<0.001*
SD, mmol/l	2.6 ± 0.6	2.6 ± 0.6	2.5 ± 0.6	1.58 (0.93, 2.68)	0.08	1.60 (0.92, 2.77)	0.09
CV%	36.0 ± 5.8	35.9 ± 5.5	36.1 ± 6.2	0.99 (0.94, 1.05)	0.83	0.99 (0.94, 1.05)	0.84
Time in target, %^a^	59.8 ± 13.3	57.6 ± 12.8	62.2 ± 13.4	0.97 (0.95, 1.00)	0.02	0.97 (0.95, 1.00)	0.04*
Time above target, %	33.7 ± 14.8	37.0 ± 13.5	30.2 ± 15.3	1.03 (1.01, 1.06)	<0.01	1.03 (1.01, 1.06)	<0.01*
Time below target, %	6.5 ± 5.6	5.4 ± 4.4	7.6 ± 6.4	0.93 (0.87, 0.98)	<0.01	0.92 (0.86, 0.98)	<0.01*
MAGE	6.2 ± 1.4	6.3 ± 1.4	6.0 ± 1.5	1.16 (0.93, 1.44)	0.18	1.16 (0.92, 1.45)	0.20
HBGI	3.3 ± 2.5	3.6 ± 2.7	2.9 ± 2.3	1.15 (1.00, 1.34)	0.04	1.15 (1.00, 1.34)	0.05
LBGI	2.6 ± 1.7	2.3 ± 1.4	2.9 ± 1.9	0.78 (0.63, 0.94)	<0.01	0.76 (0.61, 0.94)	<0.01*

Results are given as mean ± SD

^a^Defined as glucose level 3.5–7.8 mmol/l

*A significant association (*p*<0.05) in a hierarchal binary logistic regression analysis with adjustment for age, smoking, BMI and CGM device

To avoid major confounding from prematurity, we included the women with late preterm and term pregnancy in the regression analysis testing for associations with the NCO. Eight women with preterm delivery <34 weeks were excluded from the analysis. Before and after adjusting for confounders, the mean glucose levels and the percentage time spent below, in and above the target range were significantly associated with the occurrence of short-term neonatal complications in all trimesters, as was the SD of mean glucose in the third trimester (ESM Table 3).

**Fig. 2:**
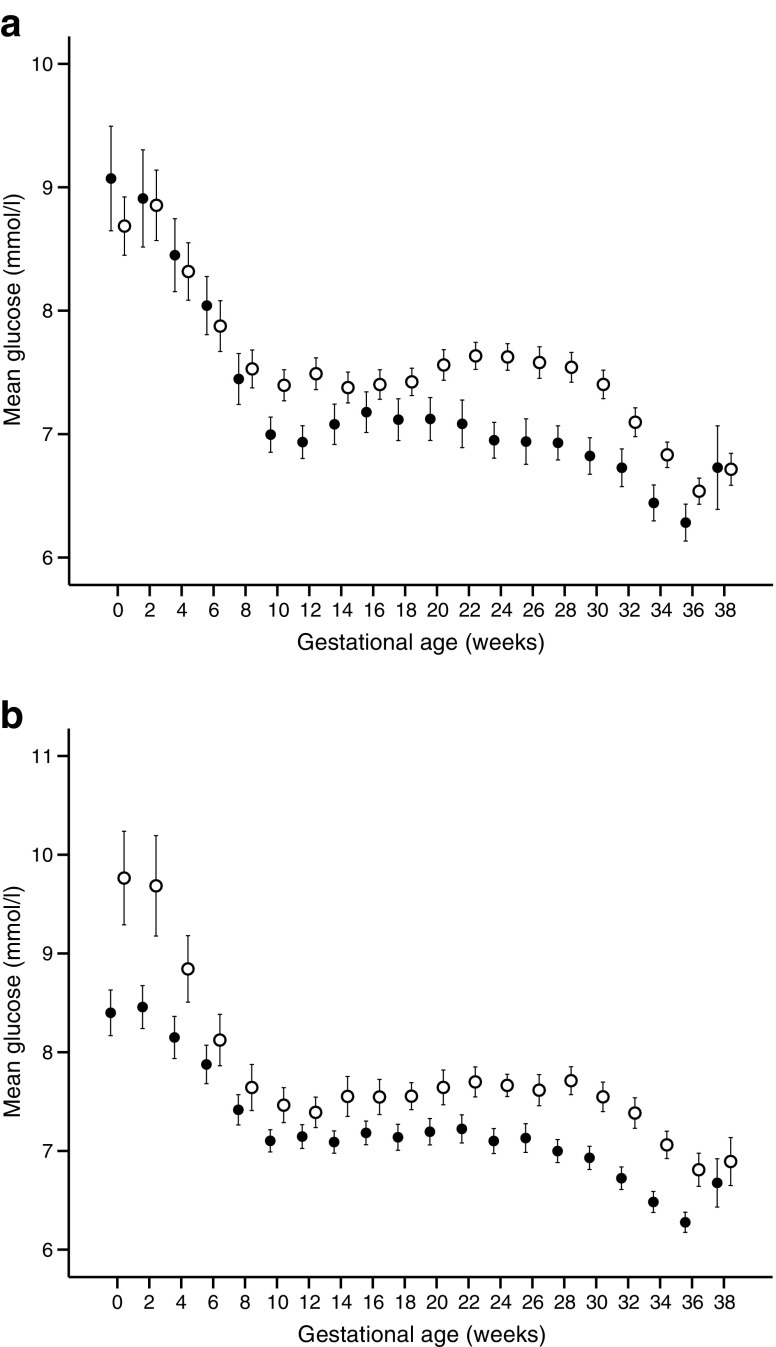
(**a**) Mean ± SEM of interstitial glucose levels during pregnancy in women with (white) and without (black) fetal overgrowth (LGA). (**b**) Mean ± SEM of interstitial glucose levels during pregnancy in women with (white) and without (black) short-term neonatal complications

As indicated in Table 3 and ESM Table 3, the LBGI values in the second and third trimesters were inversely associated with LGA (*p* = 0.01 and *p* < 0.01, respectively) and NCO (*p* < 0.01 and *p* < 0.01, respectively). Changes in mean glucose levels over gestation in relation to outcomes (LGA and NCO) in women monitored by either rtCGM or iCGM are illustrated in Fig. 2a,b. The most substantial differences in mean glucose occurred during the second and early third trimester.

## Discussion

In this study, using CGM-derived measures to describe glucose control, we found that mean glucose levels, SD of mean glucose levels, and time spent in and outside the target range (3.5–7.8 mmol/l) during the second and third trimesters were the most important predictors of LGA and neonatal outcomes. The maternal and neonatal outcomes did not differ between rtCGM users and iCGM users. The glucose patterns were comparable between the two groups throughout pregnancy, except for lower LBGI and less time spent below target in rtCGM users.

To our knowledge, this is the first study to analyse a large clinical dataset of CGM readings during pregnancy in a contemporary real-world setting. It is also the first study to report summary CGM data on the use of Freestyle Libre in pregnancies in women with type 1 diabetes. Not surprisingly, there was a clear trend of improved glucose control with increasing gestational age. The percentage of time spent in target range increased from 50% in the first trimester to 60% in the third trimester. These figures are somewhat higher than reported by Murphy et al from the first randomised controlled trial of intermittent use of CGM in pregnancy [30]. In their cohort of type 1 diabetes women, the corresponding proportions were 43% and 56%, respectively. The time spent above target in late pregnancy was similar to ours (33% vs 34%), whereas the time spent below target was higher (13% vs 7%). A more narrow definition of target range (3.9–7.8 mmol/l) may account for some of these differences. Although CGM users in the CONCEPTT study spent substantially higher time in target (68%) and less time below target (3%) compared with previous studies, the proportion of time spent above target remained high (27%) [21]. These results indicate that additional strategies might be required to optimise glucose control in pregnancies in women with type 1 diabetes―in particular to minimise postprandial glucose excursions. Closed-loop therapy in pregnancy has shown promise in reducing time in hypoglycaemia, but for now, no effect has been demonstrated on time in hyperglycaemia [31, 32].

Interestingly, we found no differences in maternal and neonatal outcomes between women using iCGM and rtCGM, which may support the non-inferior use of iCGM in pregnancy. However, the observational design of the study means that firm conclusions cannot be drawn. Of note, women using rtCGM more often used insulin pumps and had a longer duration of diabetes. Compared with iCGM users, they also spent less time in hypoglycaemia throughout pregnancy. Real-world data from Sweden suggest that insulin pump users have higher HbA_1c_ levels when starting pump therapy compared with non-pump users and are more likely to be women and aged 20–30 years [33]. Although glycaemic control measured by HbA_1c_ was similar between rtCGM users and iCGM users at baseline, this does not preclude previous differences at the time of pump therapy initiation. These circumstances mean it is likely that glycaemic disturbance in rtCGM users was more severe and these women were in greater need of a CGM system with an alarm function.

Poor glycaemic control assessed by HbA_1c_ has long been associated with accelerated fetal growth, particularly during the second and third trimesters [2, 12, 34–36]. Accordingly, in this study, HbA_1c_ was an important glucose variable, predicting LGA and neonatal outcomes―in particular, third trimester HbA_1c_. In our cohort, 36% of the women reached the target HbA_1c_ level of <48 mmol/mol (6.5%) in early pregnancy and 70% in the second and the third trimesters. These results are more favourable than those recently reported from a nationwide study in the UK, in which 16% reached the corresponding HbA_1c_ target in early pregnancy and 40% reached it after 24 weeks of gestation [15]. Nevertheless, the 53% prevalence of LGA infants is high and confirms previous findings that a substantial proportion of pregnancies among women with type 1 diabetes result in delivery of LGA infants [1–3, 21]. Our results are not directly comparable with most other studies because of differences in the definition of LGA. Using the same definition of LGA as we did (birthweight >2 SD of the ultrasound-based intrauterine reference curve), Law et al reported an LGA prevalence of 45.6% in their subgroup of 68 Danish women with pregestational diabetes randomised to intermittent use of CGM during pregnancy [18]. Taking into account that 21% of the women had type 2 diabetes, their reported LGA prevalence can be considered similar to ours. We have previously reported an LGA prevalence of 23% in pregnancies among women with type 2 diabetes, as opposed to 50% among those with type 1 diabetes [36]. Tightened glucose control early in pregnancy might possibly have changed our results. It has been argued that glycaemic control needs to be optimised very early in pregnancy to prevent fetal overgrowth as a consequence of early establishment of fetal hyperinsulinaemia, a driver of the fetal glucose steal phenomenon [37].

Given that HbA_1c_ provides a retrospective measure of average glucose levels, it is less likely to detect short-term variation in glucose levels that might be relevant in the development of LGA. However, no significant associations were found between any of the CGM measurements and LGA in the first trimester. Our data support findings from previous studies suggesting that relatively high glucose levels during the second and third trimester are predictive of LGA and adverse neonatal outcomes [12, 16, 18]. Furthermore, the SD of mean glucose in the second and third trimesters were significantly associated with LGA and NCO, respectively. Several studies have demonstrated an association between various CGM-derived measures of glucose variability and birthweight [18–20, 38]. In line with this, women in the CGM group of the CONCEPTT study had reduced SD and lower MAGE, indicating less glycaemic variability [21]. In contrast, Mulla et al did not observe any trimester-specific associations between glycaemic variability (CV%) and birthweight in a retrospective cohort study of 41 women with type 1 diabetes using real-time CGM for up to 30 consecutive days in each trimester [39]. Some of these discrepancies between studies may have arisen from differences in study design and from the use of different surrogate measures of glycaemic variability. It is important to note that the previous studies―except CONCEPTT―were based on intermittent use of CGM.

Our study should be interpreted in the context of its limitations and strengths. First, this was a clinically based observational study, which precludes us from making causal inferences. Second, the women used two different types of CGM, either rtCGM or iCGM, which may have affected the quality of glycaemic variability measurements. Third, the women were predominantly of European descent which may possibly limit the generalisability to other populations. Fourth, we followed the recently published data on use of CGM outside of pregnancy and required that there was a minimum of 14 consecutive days of data with at least 80% coverage for inclusion [22]. Considering the rapidly changing phases of insulin demands during pregnancy, 7-day profiles may better reflect the dynamic changes during pregnancy. Strengths of the study include the access to a large number of CGM readings based on optimal reports from CGM devices worn on a near-daily basis. From a clinical point of view, the observational design of the study―considering real-world data from all women using a CGM device during pregnancy―is a strength. Furthermore, information on important confounders, such as age, BMI and smoking, was available and controlled for in the logistic regression models [40].

In the present study, we sought to gain local experience of wearing CGM during pregnancy. Despite the use of CGM throughout pregnancy, the day-to-day glucose control was not optimal and the incidence of LGA remained high. There is a need for greater support from the diabetes team during pregnancy for technical assistance and intensified focus on postprandial hyperglycaemia, including dietary advice/carbohydrate counting and a supported active approach to prandial insulin adjustments. Because of ease of use and low cost, the iCGM system has become increasingly popular in Sweden among both individuals with diabetes and caregivers. The system has been considered safe and accurate for use in pregnant women with diabetes [41, 42]. It is our clinical experience that many women prefer to use iCGM rather than rtCGM in pregnancy. Further randomised trials to assess the impact of iCGM vs rtCGM on glucose control and neonatal outcomes in pregnancy are warranted.

## Electronic supplementary material

ESM Tables (PDF 231 kb)

## Data Availability

Data are available on request from the authors.

## Abbreviations

CGM: Continuous glucose monitoring
HBGI: High blood glucose index
iCGM: Intermittently viewed CGM
LBGI: Low blood glucose index
LGA: Large for gestational age
MAGE: Mean amplitude of glucose excursions
NCO: Neonatal composite outcome
NICU: Neonatal intensive care unit
rtCGM: Real-time CGM
