# Supplementary material for: Continuous glucose monitoring in pregnant women with type 1 diabetes: an observational cohort study of 186 pregnancies
Source: Diabetologia. 2019 Mar 23;62(7):1143–53. doi: 10.1007/s00125-019-4850-0 (PMC6560021; doi:10.1007/s00125-019-4850-0)
Supplement: Supplementary file 1 — (PDF 231 kb) [file 125_2019_4850_MOESM1_ESM.pdf]

ESM Table 1

Number of women and measurements available in the analysis at each trimester

	**Total, n**	**rtCGM, n**	**iCGM, n**
**All trimesters**			
Women included	186	92	94
Raw data[a] 2-week episodes	2,944	1,442	1,502
2-week episodes in analysis	2,306	986[b]	1,320[b]
Measurements in analysis	5,745,712	3,764,676	1,981,036
**Trimester 1**			
Women	155	70	85
2-week episodes	671	240	431
Measurements	1,571,461	919,413	652,048
**Trimester 2**			
Women	165	77	88
2-week episodes	955	423	532
Measurements	2,441,400	1,642,134	799,266
**Trimester 3**			
Women	167	85	82
2-week episodes	680	323	357
Measurements	1,732,851	1,203,129	529,722

[a]Including 2-week episodes with ≤ 80% coverage.
[b]The median (range) number of readings per 2-week episode was 3,862 (3,229–4,546) for women monitored with rtCGM and 1,346 (1,076–2,236) for women monitored with iCGM.

ESM Table 2 Mean glucose and glucose variability indices for women monitored by rtCGM and iCGM during pregnancy

**Gestational age (weeks)**	**1-4**	**5-8**	**9-12**	**13-16**	**17-20**	**21-24**	**25-28**	**29-32**	**33-36**	**37-40**
rtCGM (n)	22	38	59	61	68	68	73	81	66	30
iCGM (n)	49	69	71	80	80	81	81	78	72	45
**Glucose mmol/l**										
rtCGM	9.1±2.2	8.5±1.6	7.7±1.4	7.4±1.1	7.4±1.2	7.6±1.4	7.5±1.2	7.3±1.1	7.0±0.9	6.6±0.8
iCGM	8.8±1.5	8.1±1.6	7.2±1.2	7.1±1.1	7.3±1.2	7.3±1.2	7.2±1.3	7.0±1.2	6.7±1.1	6.3±0.8
**SD mmol/l**										
rtCGM	3.1±0.9	3.1±0.9	2.9±0.7	2.8±0.6	2.8±0.6	2.8±0.8	2.6±0.5	2.5±0.5	2.3±0.5	2.1±0.5
iCGM	3.6±1.0	3.3±1.0	3.0±0.9	2.9±0.8	2.8±0.7	2.7±0.7	2.6±0.6	2.4±0.6	2.3±0.6	2.2±0.6
**CV%**										
rtCGM	34.5±8.0	36.0±6.8	38.0±63.7	38.2±6.5	37.3±6.4	36.3±6.5	34.8±5.6	34.6±5.4	33.5±5.6	32.4±5.2
iCGM	40.3±6.6	40.1±7.8	41.1±8.2	40.4±7.4	38.9±6.3	37.3±6.7	36.1±6.6	34.5±5.3	34.9±5.5	35.1±6.2
**Time in target, %[a]**										
rtCGM	39.2±15.2	45.8±15.9	51.7±11.9	54.1±10.9	55.1±12.3	54.4±14.4	55.2±12.1	57.2±13.1	62.8±12.1	70.1±11.1
iCGM	42.3±16.1	47.7±16.4	54.2±15.7	54.7±14.7	54.4±14.7	55.2±15.1	56.5±16.3	59.2±15.2	63.1±13.7	68.6±11.9
**Time above target, %**										
rtCGM	58.0±16.4	50.6±18.0	41.9±15.0	38.6±13.3	38.5±14.7	39.8±16.1	39.4±14.5	37.6±15.3	32.3±13.3	25.1±12.2
iCGM	52.0±17.4	45.3±17.2	36.0±15.2	35.5±14.2	37.1±15.1	36.9±15.7	35.9±17.4	33.8±16.8	28.7±15.0	22.6±11.9
**Time below target, %**										
rtCGM	2.8±3.3	3.6±4.1	6.4±5.6	7.2±5.6	6.4±5.6	5.8±5.6	5.5±5.0	5.2±5.3	4.9±5.4	4.8±3.9
iCGM	5.6±4.9	7.0±5.3	9.8±5.5	9.8±5.8	8.6±5.5	7.8±5.8	7.6±5.4	7.0±5.4	8.2±7.3	8.7±5.8
**MAGE**										
rtCGM	8.0±2.4	7.7±2.1	7.4±1.7	7.2±1.6	7.0±1.5	7.0±1.9	6.5±1.3	6.4±1.2	6.0±1.1	5.4±1.2
iCGM	8.9±2.5	8.1±2.5	7.3±2.0	7.0±1.7	7.0±1.8	6.7±1.6	6.3±1.5	6.0±1.4	5.7±1.5	5.4±1.4
**HBGI**										
rtCGM	8.3±7.3	6.6±4.6	4.7±3.6	4.1±2.5	4.1±2.7	4.4±3.8	3.9±2.9	3.6±2.2	2.8±1.6	2.0±1.3
iCGM	7.9±4.4	6.1±4.3	4.2±2.9	3.9±2.7	4.0±2.8	3.8±3.1	3.6±2.9	3.2±2.6	2.5±2.1	1.9±1.4
**LBGI**										
rtCGM	1.1±1.1	1.4±1.3	2.4±1.7	2.6±1.7	2.4±1.8	2.2±1.6	2.1±1.6	2.1±1.7	2.1±1.7	2.3±1.3
iCGM	2.2±1.6	2.6±1.7	3.6±1.7	3.6±1.7	3.2±1.7	3.0±1.8	3.0±1.8	2.7±1.7	3.3±2.2	3.5±1.6

Results are given as mean ± SD.
[a]Defined as glucose level 3.5–7.8 mmol/L.

ESM Table 3

Results of the binary logistic regression analysis of variables tested for associations with the Neonatal Composite Outcome

	**NCO**	**No NCO**	Crude data		Adjusted data	
**Variable**	**(n=76)**	**(n=102)**	**OR (95% CI)**	***p* value**	**OR (95% CI)**	***p* value**
**Trimester 1** (n = 147)						
HbA_{1c}, mmol/mol<br>HbA_{1c}, %	55.4±12.0<br>7.2±1.1	50.0±8.9<br>6.7±0.8	1.05 (1.02–1.09)	<0.01	1.05 (1.02–1.09)	<0.01*
Mean glucose, mmol/l	8.1±1.4	7.7±1.3	1.28 (1.00-1.64)	0.05	1.29 (1.00-1.69)	<0.05
SD, mmol/l	3.3±0.9	3.0±0.8	1.52 (1.02-2.26)	0.04	1.42 (0.92-2.18)	0.11
CV%	41.1±8.0	39.6±6.4	1.03 (0.98-1.08)	0.21	1.02 (0.97-1.07)	0.54
Time in target, %[a]	46.8±14.1	52.4±13.9	0.97 (0.95-1.00)	0.02	0.97 (0.95-1.00)	0.04*
Time above target, %	46.2±15.3	40.9±15.5	1.02 (1.00-1.05)	0.04	1.03 (1.00-1.05)	0.04*
Time below target, %	7.0±5.3	6.8±4.8	1.01 (0.95-1.08)	0.79	0.99 (0.92-1.06)	0.72
MAGE	7.9±2.2	7.4±1.8	1.15 (0.97-1.37)	0.10	1.11 (0.92-1.34)	0.26
HBGI	6.2±4.1	4.9±3.3	1.10 (1.00-1.21)	0.05	1.10 (0.99-1.21)	0.60
LBGI	2.6±1.7	2.5±1.5	1.03 (0.83-1.26)	0.81	0.96 (0.76-1.20)	0.69
**Trimester 2** (n = 157)						
HbA_{1c}, mmol/mol<br>HbA_{1c}, %	47.5±8.1<br>6.5±0.7	43.6±7.5<br>6.1±0.7	1.07 (1.02–1.11)	<0.01	1.06 (1.02–1.11)	<0.01*
Mean glucose, mmol/l	7.6±0.9	7.1±1.3	1.43 (1.06-1.93)	0.01	1.48 (1.08-2.03)	0.01*
SD, mmol/l	2.8±0.6	2.7±0.6	1.49 (0.89-2.48)	0.12	1.43 (0.83-2.46)	0.20
CV%	37.3±6.7	37.6±5.6	0.99 (0.94-1.05)	0.77	0.98 (0.93-1.04)	0.49
Time in target, %[a]	51.9±12.6	57.3±13.9	0.97 (0.95-0.99)	0.01	0.97 (0.95-0.99)	0.02*
Time above target, %	41.9±12.8	35.0±15.5	1.03 (1.01-1.06)	<0.01	1.04 (1.01-1.06)	<0.01*
Time below target, %	6.2±4.7	7.7±5.2	0.94 (0.88-1.01)	0.06	0.91 (0-84-0.98)	0.01*
MAGE	6.8±1.4	6.6±1.5	1.09 (0.97-1.35)	0.45	1.06 (0.84-1.33)	0.65
HBGI	4.4±2.3	3.6±3.0	1.10 (0.98-1.24)	0.10	1.10 (0.97-1.25)	0.12
LBGI	2.3±1.4	2.9±1.7	0.79 (0.64-0.98)	0.03	0.70 (0.55-0.90)	<0.01
**Trimester 3** (n = 161)						
HbA_{1c}, mmol/mol<br>HbA_{1c}, %	48.5±7.3<br>6.6±0.7	43.9±7.2<br>6.1±0.7	1.09 (1.04-1.15)	<0.001	1.09 (1.04-1.14)	<0.00*
Mean glucose, mmol/l	7.3±0.8	6.9±1.3	1.49 (1.08-2.05)	<0.01	1.49 (1.06-2.08)	0.01*
SD, mmol/l	2.7±0.6	2.4±0.6	2.01 (1.13-3.58)	0.02	1.94 (1.06-3-55)	0.03*
CV%	36.3±6.3	35.5±5.3	1.03 (0.97-1.08)	0.39	1.02 (0.96-1.08)	0.51
Time in target, %[a]	56.4±11.0	62.5±14.1	0.96 (0.94-0.99)	<0.01	0.97 (0.94-0.99)	0.01*
Time above target, %	37.9±11.2	30.4±16.2	1.04 (1.01-1.06)	<0.001	1.04 (1.01-1.06)	<0.01*
Time below target, %	5.7±4.8	7.0±6.0	0.96 (0.90-1.02)	0.13	0.94 (0.87-1.00)	<0.05*
MAGE	6.3±1.3	6.0±1.5	1.22 (0.96-1.53)	0.09	1.20 (0.94-1.53)	0.14
HBGI	3.6±1.7	3.0±2.9	1.11 (0.97-1.28)	0.11	1.09 (0.95-1.26)	0.19
LBGI	2.2±1.4	2.8±1.9	0.80 (0.65-0.98)	0.03	0.74 (0.58-0.94)	<0.01*

Neonatal composite including ≥ 1 of the following: macrosomia (> 4,500 g), shoulder dystocia, neonatal hypoglycemia, or NICU admission > 24 hours.

Results are given as mean ± SD

[a]Defined as glucose level 3.5–7.8 mmol/l

[*]A significant association ($p<0.05$) in a hierarchal binary logistic regression analysis with adjustment for age, smoking, BMI and CGM device.
